# Population-based estimates of overtreatment with adjuvant systemic therapy in early breast cancer patients with data from the Netherlands and the USA

**DOI:** 10.1007/s10549-022-06550-2

**Published:** 2022-03-03

**Authors:** M. A. A. Ragusi, B. H. M. van der Velden, M. C. van Maaren, E. van der Wall, C. H. van Gils, R. M. Pijnappel, K. G. A. Gilhuijs, S. G. Elias

**Affiliations:** 1 Department of Radiology/Image Sciences Institute, University Medical Center Utrecht, Utrecht University, Heidelberglaan 100, 3584 CX Utrecht, The Netherlands; 2 Department of Research and Development, Netherlands Comprehensive Cancer Organisation, Godebaldkwartier 419, 3511 DT Utrecht, The Netherlands; 3 Department of Health Technology and Services Research, Technical Medical Centre, University of Twente, Hallenweg 5, 7522 NH Enschede, The Netherlands; 4 Department of Medical Oncology, University Medical Center Utrecht, Utrecht University, Heidelberglaan 100, 3584 CX Utrecht, The Netherlands; 5 Department of Epidemiology, Julius Center for Health Sciences and Primary Care, University Medical Center Utrecht, Utrecht University, Universiteitsweg 100, 3584 CG Utrecht, The Netherlands

**Keywords:** Breast cancer, Overtreatment, Chemotherapy, Endocrine therapy, Targeted therapy

## Abstract

**Purpose:**

Although adjuvant systemic therapy (AST) helps increase breast cancer-specific survival (BCSS), there is a growing concern for overtreatment. By estimating the expected BCSS of AST using PREDICT, this study aims to quantify the number of patients treated with AST without benefit to provide estimates of overtreatment.

**Methods:**

Data of all non-metastatic unilateral breast cancer patients diagnosed in 2015 were retrieved from cancer registries from The Netherlands and the USA. The PREDICT tool was used to estimate AST survival benefit. Overtreatment was defined as the proportion of patients that would have survived regardless of or died despite AST within 10 years. Three scenarios were evaluated: actual treatment, and recommendations by the Dutch or USA guidelines.

**Results:**

59.5% of Dutch patients were treated with AST. 6.4% (interquartile interval [IQI] = 2.5, 8.2%) was expected to survive at least 10 years due to AST, leaving 93.6% (IQI = 91.8, 97.5%) without AST benefit (overtreatment). The lowest expected amount of overtreatment was in the targeted and chemotherapy subgroup, with 86.5% (IQI = 83.4, 89.6%) overtreatment, and highest in the only endocrine treatment subgroup, with 96.7% (IQI = 96.0, 98.1%) overtreatment. Similar results were obtained using data from the USA, and guideline recommendations.

**Conclusion:**

Based on PREDICT, AST prevents 10-year breast cancer death in 6.4% of the patients treated with AST. Consequently, AST yields no survival benefit to many treated patients. Especially improved personalization of endocrine therapy is relevant, as this therapy is widely used and is associated with the highest amount of overtreatment.

**Supplementary Information:**

The online version contains supplementary material available at 10.1007/s10549-022-06550-2.

## Introduction

Adjuvant systemic treatment (AST) has contributed to a reduction of breast cancer mortality over the past decades [1, 2]. Whether a patient is recommended AST, and if so what type (endocrine, targeted, chemotherapy, or a combination) differs between countries but largely depends on several clinicopathological variables, including patient age, receptor status, tumor extent, tumor grade, and axillary tumor load. For example, the Dutch guidelines recommends AST when the absolute 10-year breast cancer-specific survival (BCSS) is expected to increase by at least 3% [3]. Such BCSS-gain depends on clinicopathological variables and can be estimated for individual patients with tools such as PREDICT [4–6], which is endorsed by the Dutch breast cancer guidelines as well as the American Joint Committee on Cancer (AJCC) [6, 7].

Over time, AST recommendations have expanded to include more favorable prognostic subgroups [8]. For example, only 23% of all breast cancer patients received endocrine therapy and 11% chemotherapy in 1990 in the Netherlands [8], which increased to 56% and 44%, respectively, by 2012 [8]. Parallel to this trend, there is a growing concern about overtreatment.

Patients treated with AST but without benefit, because they would have survived breast cancer also without AST, or because they died from breast cancer despite AST, can be considered overtreated [9, 10]. Such patients are unnecessarily exposed to the adverse effects of AST on health and quality of life [11]. Additionally, overtreatment also leads to unnecessary health care and societal costs.

Estimates of overtreatment can directly be derived from randomized controlled trials, but such studies often do not reflect everyday clinical practice with regard to patient mix and treatment standardization [12–14]. To address and substantiate the growing concern about AST overtreatment, there is, therefore, a need for population-based estimates of overtreatment associated with contemporary real-world AST prescribing practice. Such estimates are currently lacking.

In this study we aimed to estimate the amount of AST overtreatment, overall and separately for endocrine, targeted, and chemotherapy, on a population level in real-world clinical care. For this we used population-based data from the Netherlands and the United States of America (USA) of breast cancer patients diagnosed in 2015. To obtain estimates of overtreatment, we projected individual BCSS-gain over a 10-year horizon using PREDICT, which we aggregated for all patients actually treated, or recommended to be treated with AST based on the Dutch or USA guidelines. Development and use of tools aimed at curbing overtreatment will be most relevant in breast cancer patients in whom the magnitude of overtreatment is particularly high.

## Methods

### Design

This study used real-world observational data from population-based cohorts of patients diagnosed with breast cancer in 2015 from two cancer registries: the Netherlands Cancer Registry (NCR) and the Surveillance, Epidemiology, and End Results (SEER) Program from the USA. In real-world observational data, estimates of overtreatment cannot be directly observed as it is impossible to distinguish whether a treated breast cancer patient survived because of AST or would also have survived without AST. Overtreatment estimates in the context of breast cancer survival using observational data can, however, be obtained by summarizing predictions of BCSS-gain by AST per patient. In this study we used PREDICT (version 2.0) to obtain such estimates of BCSS-gain from AST [4–6]. PREDICT is an algorithm that uses several patient-specific clinicopathological variables to predict the absolute risk of dying from breast cancer over a 10-year horizon in the absence of AST, and then projects the therapeutic BCSS-gain of different AST subtypes as derived from randomized clinical trials to obtain an estimate of absolute individual BCSS-gain due to specific types of AST [4–6]. PREDICT performs well in many different prognostic subgroups and accurately projects absolute BCSS, adjusted for competing causes of death, in the presence and absence of administered AST [6, 15–18].

In this study, we address both overtreatment due to actual AST use as well as guideline-recommended AST use. Estimates of overtreatment due to actual AST use were based on patients registered by the NCR to have been treated with AST, which included type of treatment (i.e., endocrine, targeted, or chemotherapy, as mono- or combination therapy). As actual AST use is unavailable from SEER [19], we were unable to investigate actual AST use in the USA. To investigate overtreatment associated with guideline recommendations we applied both the Dutch (version 2.0) [3] and the National Comprehensive Cancer Network (NCCN) guidelines (version 3.2015) [20] to both the Dutch and USA cohorts. Both guidelines were applied to both cohorts because the distribution of clinicopathological variables (i.e., the patient mix) may differ between countries (e.g., due to different breast screening strategies), which could lead to different expected BCSS-gain from AST on a population level.

### Patient data

From the Dutch cohort we obtained all patient, tumor, and treatment characteristics of all female non-metastatic breast cancer patients diagnosed in 2015 (*N* = 15,007). Patients who did not receive surgery (*N* = 1082), who received neoadjuvant treatment (*N* = 2926), or patients with bilateral tumors (*N* = 189) were excluded, leaving a total of 10,810 patients for analysis. Similarly, from the USA cohort we obtained all patient, and tumor characteristics of all female non-metastatic breast cancer patients diagnosed in 2015 (*N* = 58,429)[21]. Patients without data available from surgical pathology (*N* = 11,214), who received neoadjuvant treatment (*N* = 481, based on pathological staging), or patients with bilateral tumors (*N* = 981) were also excluded, leaving a total of 45,753 patients for analysis.

### AST guidelines

We applied the 2012 Dutch guidelines (version 2.0, pertinent in 2015) [3] to both the Dutch and USA cohort. Similarly we applied the USA 2015 guidelines (version 2015.3) [20] to both the Dutch and USA cohort (Supplemental Materials 1 shows an overview of the differences between these guidelines). Some adaptions and interpretations of these guidelines were necessary. First, we did not have the results of any possibly performed genomic assays available, and did, therefore, not take this into account. Second, when the guidelines were ambiguous, we applied the strictest recommendations. For instance, although the USA guidelines states to consider adjuvant endocrine therapy in a node-negative ER+/HER2− tumor of size ≤ 5 mm, we analyzed the data considering endocrine therapy to be not recommended in these patients.

### Estimation of BCCS-gain and overtreatment from AST

**Fig. 1:**
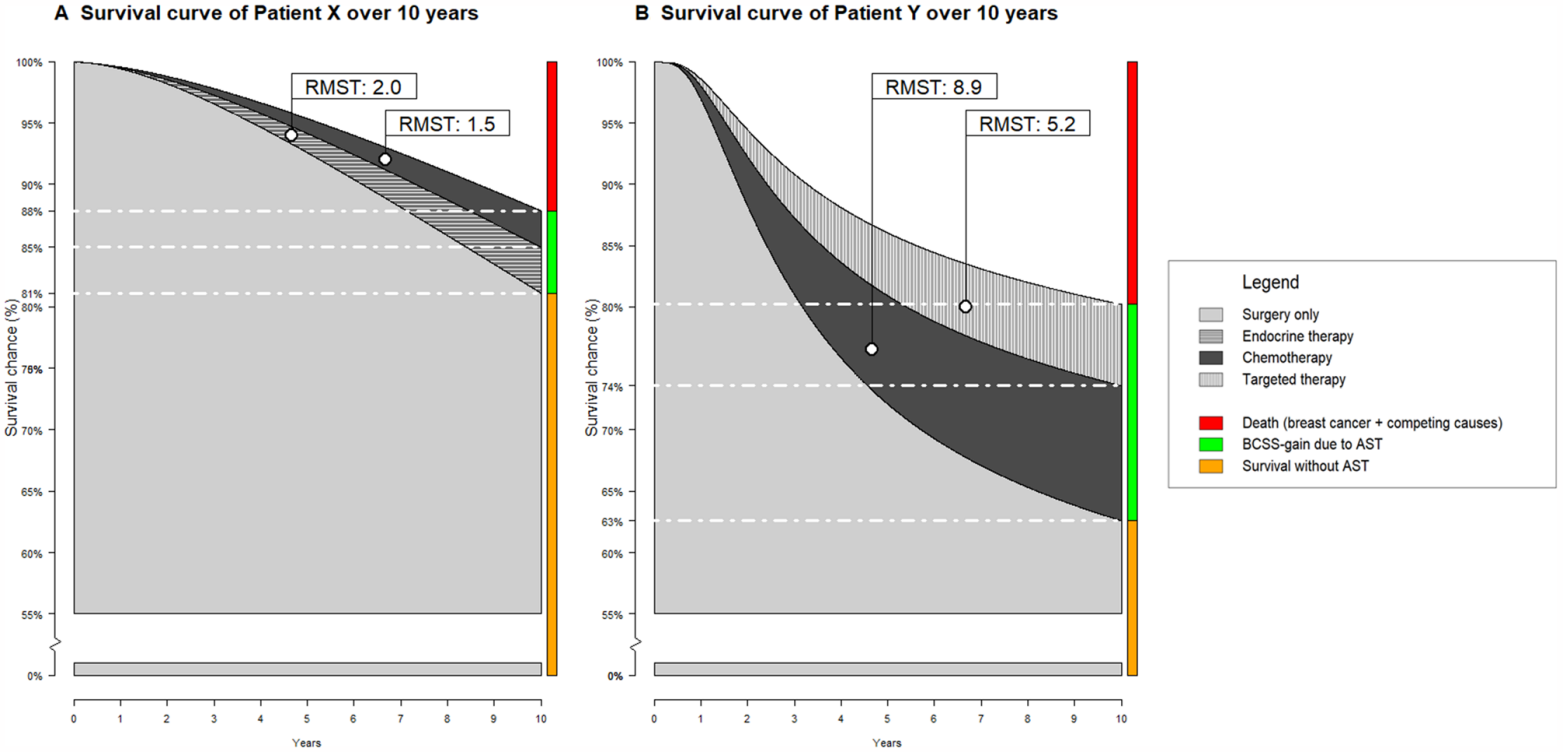
Two predicted 10-year survival curves of hypothetical patient X (**A**) and patient Y (**B**) estimated by the PREDICT algorithm with and without AST. A. The left survival curve (patient X) represents a 57-year old patient with a grade 2 ER+/HER2− tumor with a size of 29 mm and no positive lymph nodes (pT2N0). Without AST this patient is expected to have an 81% chance at surviving for at least 10 years. After treatment with adjuvant endocrine therapy this would increase to 85% (an additional 4%), and with adjuvant chemotherapy this would further increase to 88% (an additional 3%), for a total expected increase in 10-year BCSS of 7% for the combination treatment. Overtreatment is calculated as the proportion of patients that would have survived for at least 10 years regardless of AST or died despite AST, e.g.: if 100 Patient X’s were to be treated then we would expect 7 patients to have survived longer due to AST, while 81 patients would have survived regardless of AST and 12 patients would have died despite AST, therefore, 93 patients are overtreated. The expected survival gain expressed in the additional months of survival over a 10 year horizon, i.e., the RMST, would be 2 months for endocrine therapy alone, and 1.5 months for chemotherapy alone, amounting to an additional 3.5 months of survival within the first 10 years. Without AST this patient would have an estimated 110.4 months (9.2 years) of survival over a 10 year horizon. B. The right survival curve (patient Y) represents a 38-year old patient with a grade 3 ER−/HER2+ tumor with a size of 18 mm and 1 positive lymph node. Patient Y has a 63% chance of at least 10-year survival (in the absence of treatment with AST), this would increase by 11% with chemotherapy (to 74% 10-year survival), and additional 6% when treated with targeted therapy (to a total of 80% survival), for a total expected increase in 10-year BCSS of 17% for the combination treatment. Similar to the example described in A, this would lead to an overtreatment of 83% of patients. The RMST would increase by 8.9 months when treated with chemotherapy and by 5.2 months when treated with targeted therapy, amounting to a total of 14.2 months of additional survival within the first 10-years. Without AST this patient would have an estimated 91.3 months (7.6 years) of survival over a 10 year horizon. *RMST* restricted mean survival time, *BCSS* breast cancer-specific survival, *AST* adjuvant systemic treatment, *ER* estrogen receptor, *HER2* human epidermal growth factor receptor-2

PREDICT (version 2.0) estimates BCSS over a 10-year horizon from the different subtypes of AST based on several patient and tumor characteristics. PREDICT takes the following characteristics as input: age, mode of detection, tumor size, tumor grade, number of positive lymph nodes, ER and HER2 status, Ki67 status and chemotherapy generation. Ki67 status is not registered in the Dutch or the USA cohort and was always coded as unknown. A PREDICT script was created to calculate predicted 10-year BCSS-gain from each AST subtype. Additionally, the PREDICT script was adapted to calculate the area under the curve (AUC) of patient-specific predicted survival curves in the absence and presence of AST for the calculation of 10-year restricted mean survival time (RMST). RMST is the mean of the time to an event limited to some ‘horizon’ time (e.g., 10 years) [22]. It equals the AUC of the survival curve to that point in time [22]. The increased RMST due to AST can be interpreted as the added average survival time (or time to event) due to AST within these 10 years (for further explanation see Fig. 1) [22, 23].

To estimate the amount and distribution of expected overtreatment, we calculated the 10-year BCSS-gain, numbers needed to treat (NNT), and RMST (total and per patient) from AST based on actual treatment as registered in the Netherlands and the recommended treatment based on the Dutch and USA guidelines in both the Netherlands and USA. We defined overtreatment as the proportion of patients who would have survived without AST or died despite AST until the 10-year mark (Fig. 1). Overtreatment per patient was calculated by adding the probability that this patient would have survived regardless of AST (the orange section in Fig. 1) or died despite AST (the red section in Fig. 1) at the 10-year mark. The patient-specific BCSS-gain was calculated by adding the BCSS-gain from the individual subtypes of AST that was received by or recommended to a patient, e.g., if a patient received both endocrine and chemotherapy the total BCSS-gain was calculated as the BCSS-gain from endocrine therapy plus chemotherapy (the green section in Fig. 1). The numbers needed to treat (NNT) was calculated as the reciprocal of the total BCSS-gain (i.e., 1/BCSS-gain). To calculate the population-based distribution of overtreatment and BCSS-gain, these estimates were aggregated over treatment groups (endocrine, targeted, and chemotherapy). Treatment-specific BCSS-gain was aggregated for all received or recommended AST because treatment decisions are based on total BCSS-gain, i.e., the Dutch guidelines recommend (combination) AST when the total BCSS-gain is ≥ 3% [3]. To quantify the number of patients experiencing low predicted BCSS-gain, we set a threshold of < 3% total BCSS-gain from AST [3].

### Statistical analysis

Missing variables of interest were multiply-imputed [24]. The number of imputed datasets was based on the percentage of rows with a missing variable of interest (20% in the Dutch cohort, and 25% in the USA cohort). Multiply-imputed estimates were aggregated using Rubin’s Rules [25]. Estimates of (aggregated) overtreatment are reported as the mean, whereas BCSS-gain, NNT, and RMST are reported as median with their corresponding interquartile interval (IQI). Statistical analyses were performed using R version 3.6.2 (R Foundation for Statistical Computing, Vienna, Austria) and the multiple imputation was performed using the ‘mice’ (version 3.8.0) [26] package available in R.

## Results

**Fig. 2:**
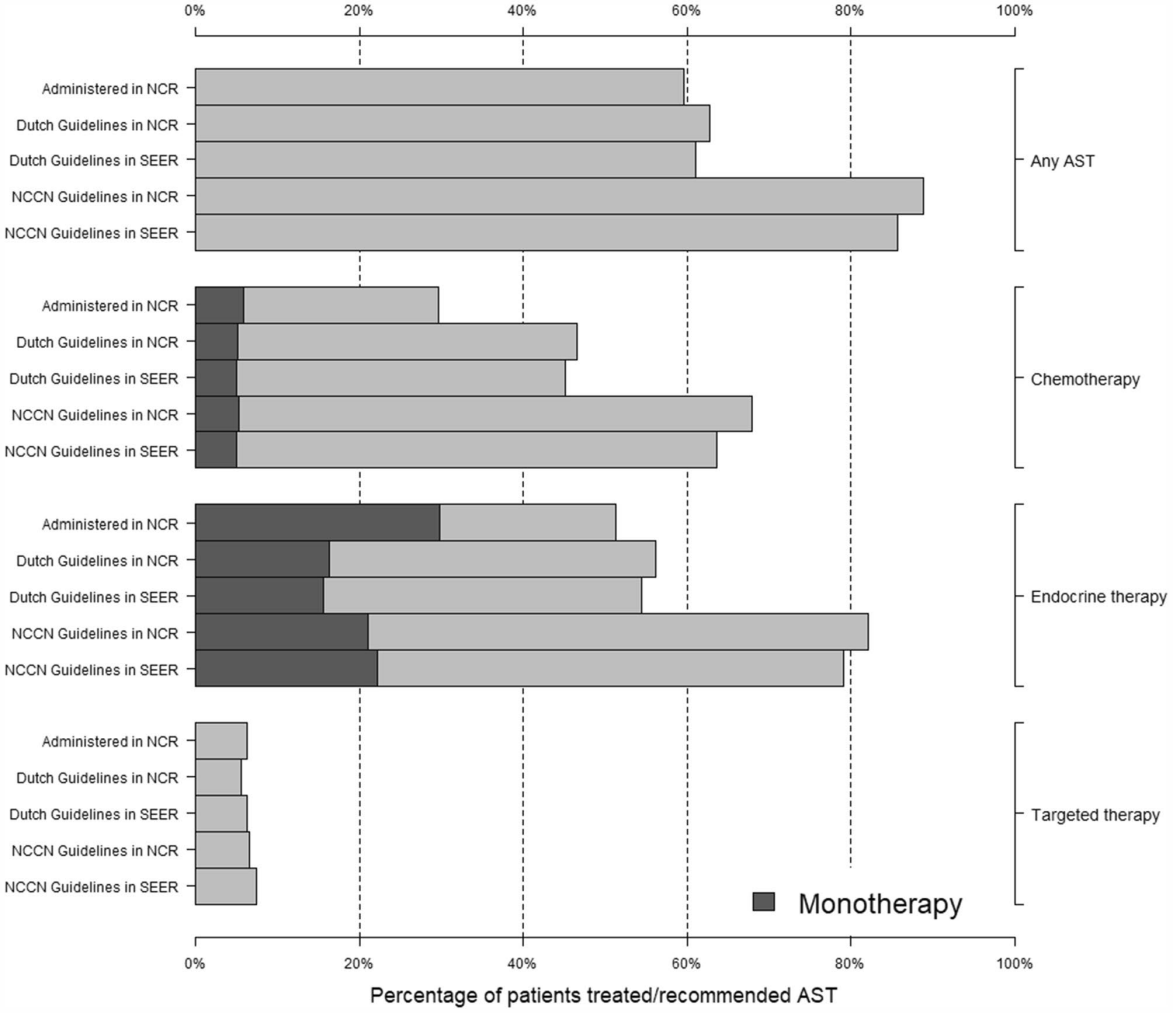
The distribution of administered and recommended AST, overall and according to subtype, for all surgically treated unilateral non-metastatic breast cancer patients in the Netherlands (NCR) and the USA (SEER) in 2015. Recommendations are based on the 2015 Dutch and USA guidelines. Patients who are treated with monotherapy (a single type of AST) are also indicated. *AST* adjuvant systemic therapy, *NCR* Netherlands Cancer Regsistry, *SEER* Surveillance, Epidemiology, and End Results Program, *NCCN* National Comprehensive Cancer Network

Table 1 shows the distribution of clinicopathological variables at diagnosis for both the Netherlands (*N* = 10,810) and the USA (*N* = 45,753). The median patient age was 63 years (IQI = 53, 71) in both cohorts. Overall, baseline clinicopathological variables were similar between the Netherlands and the USA. The frequency of actual AST distribution in the Netherlands, and AST recommendations based on the Dutch and USA guidelines is shown in Fig. 2. Overall, Dutch early breast cancer patients received less chemotherapy than indicated based on the guidelines, particularly because a large proportion of patients with an indication for both endocrine therapy and chemotherapy, were actually treated with monoendocrine therapy. Compared to the Dutch guidelines, The USA recommends chemotherapy and endocrine therapy to a larger proportion of patients.

**Table 1:** Characteristics of all female patients surgically treated for unilateral non-metastatic breast cancer without neoadjuvant therapy in 2015 in NL and USA

	NL (*N* = 10,810)	USA (*N* = 45,753)
*Age (years)*		
Median (IQI)	63 (53, 71)	63 (53, 71)
≤ 39	279 (3%)	1443 (3%)
40–49	1289 (12%)	6163 (13%)
50–74	7679 (71%)	30,052 (66%)
75–84	1282 (12%)	6346 (14%)
≥ 85	281 (3%)	1749 (4%)
*Tumor size (mm)*		
Median (IQI)	15 (10, 22)	15 (9, 23)
≤5	758 (7%)	5006 (11%)
6–10	2139 (20%)	9329 (20%)
11–20	4866 (45%)	17,463 (38%)
21–50	2740 (25%)	12,203 (27%)
> 50	308 (3%)	1751 (4%)
*Number of positive lymph nodes*		
0	7945 (73%)	33,331 (73%)
1–3	2438 (23%)	9890 (22%)
3–9	271 (3%)	1773 (4%)
≥ 10	156 (1%)	759 (2%)
*Tumor grade*		
1	3006 (28%)	12,674 (28%)
2	5262 (49%)	21,528 (47%)
3	2542 (24%)	11,551 (25%)
*IHC-subtype*		
ER+/HER2+	721 (7%)	4014 (9%)
ER+/HER2−	8754 (81%)	36,142 (79%)
ER−/HER2+	329 (3%)	1555 (3%)
TN	1006 (9%)	4042 (9%)

Unless otherwise specified, data are number of patients, with percentages between parentheses. Data are after multiple imputation

*NL* Netherlands, *USA* United States of America, *IQI* interquartile interval, *IHC* immunohistochemical, *ER* estrogen receptor, *HER* human epidermal growth factor-2, *TN* triple-negative

### Overtreatment estimates of AST using actual prescribed treatment in the Netherlands

Table 2 shows the expected population-level 10-year overtreatment and survival benefit of each of the actually administered AST subtypes and regimens in the Netherlands. Overall, a total of 6431 patients (59.5%) received any type of AST in the Dutch cohort in 2015. AST (any combination) is expected to save 409 patients (6.4%) from dying of breast cancer within 10 years. The remaining 6022 patients (93.6%) are expected to be unaffected, i.e., overtreated, because 4509 patients (70.1%) are expected to survive also in absence of AST, and 1513 patients (23.5%) are expected to die from breast cancer or other causes despite AST. The median estimated 10-year absolute BCSS-gain in those treated with AST is 4.7% (IQI = 2.5, 8.2%), equivalent to an NNT of 21.4 (IQI = 12.1, 40.5) for patients who received any combination of AST (Table 2). The aggregated amount of expected increased survival time within the first 10 years due to AST (i.e., total RMST) was 2105.5 years for the entire Dutch population treated with AST, or 3.9 months (IQI = 1.3, 5.2) per patient.

**Table 2:** Expected survival benefit of administered AST regimens in female patients surgically treated for unilateral non-metastatic breast cancer without neoadjuvant therapy in 2015 in the Netherlands (*n* = 10,810)

AST regimen	Number of patients treated (% of total)	Median Expected 10-year BCSS-gain (IQI)	Expected number of patient outcomes over a 10-year horizon			Expected NNT to prevent one breast cancer death in 10 years (IQI)	Total increased RMST in years from AST within 10 years	Median increased RMST in months from AST within 10 years per patient (IQI)	Number of treated patients with < 3% 10-year BCSS-gain (% of treated)
			Benefit from AST	No benefit from AST, i.e., overtreatment					
			Survived due to AST (%)	Survived not due to AST (%)	Died despite AST (%)				
Any*	6431 (59.5%)	4.7 (2.5, 8.2)	409 (6.4%)	4509 (70.1%)	1513 (23.5%)	21.4 (12.1, 40.5)	2105.5	3.9 (1.3, 5.2)	2104 (32.7%)
*Chemotherapy*									
Monotherapy	627 (5.8%)	7.0 (5.9, 8.6)	45 (7.3%)	428 (68.2%)	154 (24.5%)	14.3 (11.7, 16.9)	297.1	5.7 (4.4, 6.7)	52 (8.3%)
In combination**	2577 (23.8%)	8.3 (5.1, 13.0)	257 (10.0%)	1896 (73.6%)	424 (16.4%)	12.0 (7.7, 19.5)	1288.1	6.0 (2.7, 7.6)	130 (5.0%)
Contribution to combination***	2577 (23.8%)	3.7 (2.2, 6.1)	119 (4.6%)	1896 (73.6%)	562 (21.8%)	27.3 (16.5, 45.1)	600.2	2.8 (1.1, 3.3)	1025 (39.8%)
*Endocrine therapy*									
Monotherapy	3213 (29.7%)	2.6 (1.9, 4.0)	105 (3.3%)	2177 (67.7%)	931 (29.0%)	38.7 (24.7, 52.4)	514.8	1.9 (1.0, 2.2)	1918 (59.7%)
In combination**	2326 (21.5%)	7.9 (4.9, 12.2)	223 (9.6%)	1740 (74.8%)	363 (15.6%)	12.6 (8.1, 20.1)	1062.0	5.5 (2.6, 6.7)	124 (5.3%)
Contribution to combination***	2326 (21.5%)	4.3 (2.7, 6.3)	114 (4.9%)	1740 (74.8%)	472 (20.3%)	23.3 (15.8, 37.2)	553.0	2.9 (1.4, 3.5)	724 (31.1%)
Targeted therapy****									
In combination**	678 (6.3%)	12.1 (7.5, 16.5)	88 (12.9%)	462 (68.2%)	128 (18.9%)	8.2 (6.0, 13.2)	483.7	8.5 (4.0, 11.1)	21 (3.1%)
Contribution to combination***	678 (6.3%)	2.9 (1.6, 4.9)	25 (3.7%)	462 (68.2%)	191 (28.1%)	34.1 (20.2, 60.5)	140.3	2.5 (0.9, 3.6)	344 (50.7%)
*Specific combinations*									
Endocrine, targeted, and chemotherapy	408 (3.8%)	10.9 (6.8, 16.4)	52 (12.7%)	295 (72.4%)	61 (15.0%)	9.1 (6.1, 14.5)	246.7	7.2 (3.5, 9.1)	10 (2.5%)
Endocrine and chemotherapy	1907 (17.6%)	7.4 (4.7, 11.2)	171 (8.9%)	1438 (75.4%)	298 (15.6%)	13.5 (8.9, 21.1)	809.9	5.1 (2.4, 6.1)	111 (5.8%)
Targeted and chemotherapy	259 (2.4%)	13.1 (10.4, 16.6)	35 (13.5%)	160 (61.9%)	64 (24.6%)	7.6 (6, 9.6)	231.5	6.5 (1.6, 10.3)	10 (3.9%)

Data are given as median (IQI), unless specified otherwise

*AST* adjuvant systemic therapy, *BCSS* breast cancer-specific survival, *NNT* number needed to treat, *RMST* restricted mean survival time

*Endocrine, targeted, and/or chemotherapy

**Total BCSS-gain from all AST in the combination therapy (monotherapy is not included)

***Contribution of AST subtype to the combination therapy

****Only one patient was treated with monotargeted therapy, therefore, this row is omitted

A relatively large proportion of patients (who were recommended endocrine and chemotherapy, but only received endocrine therapy) received a different AST regimen compared to the guideline recommendations (*N* = 1606, Fig. 2). The median age of this subgroup was higher compared to the subgroup of patients who did receive endocrine and chemotherapy: 62 (IQI = 44, 70) versus 54 (IQI = 37, 68). The expected overtreatment was 97.2% (IQI = 97.0, 98.2%) based on the treatment they received (monoendocrine therapy) as opposed to an expected overtreatment of 95.0% (IQI = 94.6, 96.8%) based on the treatment they were recommended (endocrine and chemotherapy).

**Fig. 3:**
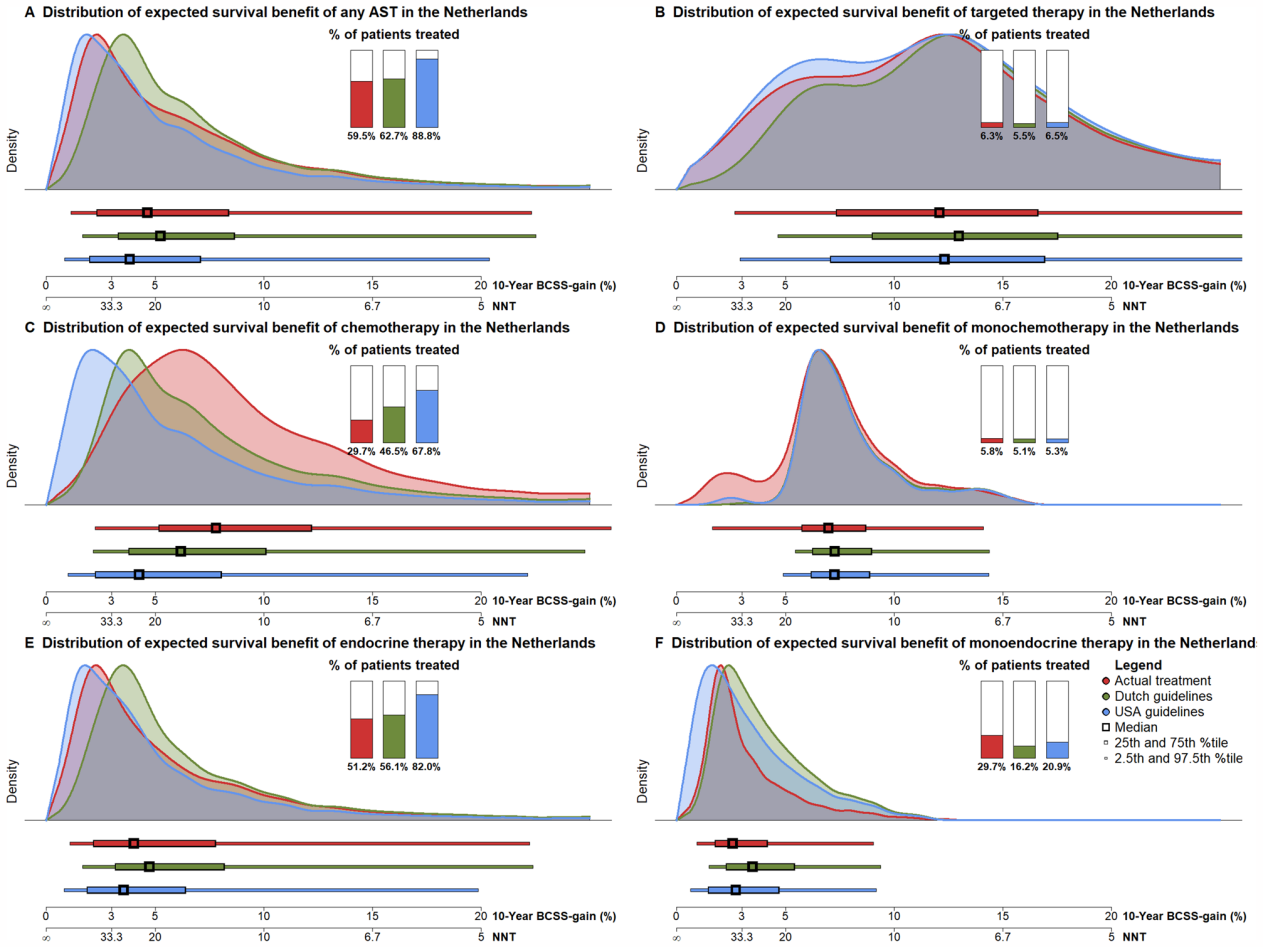
The distribution of expected 10-year BCSS-gain and NNT for all patients in the Netherlands who received or were recommended any AST (**A**), who were treated with targeted therapy (**B**), chemotherapy (**C**), monochemotherapy (**D**), endocrine (**E**) and monoendocrine therapy (**F**). For the overall AST subgroups (i.e., **A**, **B**, **C**, and **E**) the total BCSS-gain is aggregated from each received or recommended AST, e.g., chemotherapy (**C**) shows the distribution of total BCSS-gain of the entire registered or recommended AST regimen (including targeted and/or endocrine therapy) of patients that received chemotherapy (including monochemotherapy). Although the proportion of patients treated with endocrine therapy based on actual registered treatment and recommendations based on the Dutch guidelines is similar, the average survival benefit based on the Dutch guidelines is higher. This is due to the fact that a large proportion of patients that are recommended endocrine and chemotherapy, were actually treated with monoendocrine therapy, leading to a lower expected increased survival benefit (but also less treatment). Note that only one patient was registered with monotargeted therapy and guidelines do not recommend treatment with monotargeted therapy, therefore, the distribution is unavailable. *AST* adjuvant systemic treatment, *BCSS* breast cancer-specific survival, *NNT* number needed to treat, *USA* United States of America

Patients who were treated with monoendocrine therapy were expected to experience a high probability of overtreatment and low BCSS-gain. Treatment with monoendocrine therapy of 3213 (29.7% of all breast cancer patients) resulted in an expected overtreatment of 96.7%. Figure 3 shows the distribution of 10-year BCSS-gain for the different treatment regimens based on actual treatment but also based on Dutch and USA guideline treatment recommendations.

### Overtreatment estimates AST based on guideline recommendations in the Netherlands and the USA

Table 3 shows the expected population-level overtreatment and 10-year survival benefit of each of the recommended AST subtypes and regimens in Dutch patients based on Dutch and USA guidelines. Overtreatment was expected to be higher when based on USA guidelines compared to Dutch guidelines: 94.5% vs 93.1% of patients were overtreated in the any AST subgroup. The distribution of expected survival benefit of the different AST regimens based on Dutch and USA guidelines is shown in Fig. 3. Overall, the USA recommended endocrine and chemotherapy to a larger number of patients (with a more favorable prognostic profile), resulting in lower survival benefit for these patients. Similarly, these analyses were applied to the patients from the USA (Supplemental Materials 2 and 3).

**Table 3:** Expected survival benefit of recommended AST regimens based on Dutch and USA guidelines in female patients surgically treated for unilateral non-metastatic breast cancer without neoadjuvant therapy in 2015 in the Netherlands (*N* = 10,810)

AST regimen	Number of patients treated (% of total)	Median expected 10-year BCSS-gain (IQI)	Expected number of patient outcomes over a 10-year horizon			Expected NNT to prevent one breast cancer death in 10 years (IQI)	Total increased RMST in years from AST within 10 years (years)	Median increased RMST in months from AST within 10 years per patient (IQI)	Number of treated patients with < 3% 10-year BCSS-gain (% of treated)
			Benefit from AST	No benefit from AST, i.e., overtreatment					
			Survived due to AST (%)	Survived not due to AST (%)	Died despite AST (%)				
Any*									
NL	6782 (62.7%)	5.3 (3.5, 8.5)	470 (6.9%)	4678 (69.0%)	1634 (24.1%)	19.0 (11.7, 28.9)	2378.7	4.2 (1.8, 5.3)	1155 (17.0%)
USA	9595 (88.8%)	3.8 (2.1, 6.9)	526 (5.5%)	6990 (72.8%)	2079 (21.7%)	26.0 (14.4, 47.0)	2622.1	3.3 (1.1, 4.2)	3697 (38.5%)
*Chemotherapy*									
Monotherapy									
NL	554 (5.1%)	7.3 (6.4, 8.8)	44 (8.0%)	370 (66.7)	140 (25.3%)	13.8 (11.3, 15.7)	299.9	6.5 (5.0, 7.0)	0 (0.0%)
USA	568 (5.3%)	7.3 (6.3, 8.7)	45 (7.9%)	381 (67.0%)	143 (25.1%)	13.8 (11.5, 15.8)	302.0	6.4 (4.9, 6.9)	10 (1.8%)
In combination**									
NL	4471 (41.4%)	5.7 (3.8, 10.2)	354 (7.9%)	3444 (77.0%)	673 (15.1%)	17.5 (9.8, 26.5)	1705.1	4.6 (1.9, 5.5)	456 (10.2%)
USA	6757 (62.5%)	4.0 (2.2, 7.7)	405 (6.0%)	5415 (80.1%)	937 (13.9%)	25.3 (13, 44.4)	1926.9	3.4 (1.1, 4.1)	2452 (36.3%)
Contribution to combination***									
NL	4471 (41.4%)	2.5 (1.7, 4.4)	160 (3.6%)	3444 (77.0%)	867 (19.4%)	40.2 (22.7, 60.1)	772.9	2.1 (0.8, 2.3)	2639 (59.0%)
USA	6757 (62.5%)	1.7 (1.0, 3.3)	182 (2.7%)	5415 (80.1%)	1160 (17.2%)	57.7 (30.1, 101.4)	868.7	1.5 (0.5, 1.7)	4876 (72.2%)
*Endocrine therapy*									
Monotherapy									
NL	1751 (16.2%)	3.5 (2.4, 5.3)	71 (4.1%)	861 (49.1%)	819 (46.8%)	28.6 (18.9, 41.2)	373.8	2.6 (1.3, 3.1)	695 (39.7%)
USA	2264 (20.9%)	2.7 (1.6, 4.6)	76 (3.4%)	1190 (52.6%)	998 (44.1%)	36.7 (21.8, 62.1)	393.2	2.1 (0.8, 2.6)	1236 (54.6%)
In combination**									
NL	4312 (39.9%)	5.5 (3.7, 9.5)	328 (7.6%)	3351 (77.7%)	633 (14.7%)	18.1 (10.5, 26.8)	1526.1	4.2 (1.9, 5.1)	456 (10.6%)
USA	6595 (61.0%)	3.9 (2.2, 7.1)	378 (5.7%)	5321 (80.7%)	896 (13.6%)	25.8 (14, 45.2)	1741.4	3.2 (1.1, 3.8)	2452 (37.2%)
Contribution to combination***									
NL	4312 (39.9%)	3.0 (2.1, 5)	171 (4.0%)	3351 (77.7%)	790 (18.3%)	33.5 (19.8, 48.1)	807.4	2.2 (1.1, 2.7)	2167 (50.3%)
USA	6595 (61.0%)	2.1 (1.2, 3.8)	198 (3.0%)	5321 (80.7%)	1076 (16.3%)	46.6 (25.9, 80.5)	925.4	1.7 (0.6, 2.7)	4356 (66.1%)
Targeted therapy****									
In combination**									
NL	592 (5.5%)	13.0 (9.1, 17.4)	84 (14.2%)	406 (68.5%)	102 (17.3%)	7.7 (5.7, 10.9)	455.9	9.2 (4.9, 11.7)	3 (0.5%)
USA	698 (6.5%)	12.0 (6.4, 16.5)	92 (13.1%)	489 (70.1%)	117 (16.8%)	7.2 (4.5, 10)	490.5	8.4 (3.8, 11.1)	18 (2.6%)
Contribution to combination***									
NL	592 (5.5%)	3.0 (1.8, 5.1)	22 (3.8%)	406 (68.5%)	164 (27.7%)	33.6 (19.7, 54.4)	124.8	2.5 (0.9, 3.7)	297 (50.2%)
USA	698 (6.5%)	2.6 (1.4, 4.9)	24 (3.5%)	489 (70.1%)	185 (26.4%)	38.9 (20.5, 69.3)	132.9	2.3 (0.7, 3.5)	395 (56.6%)
Specific combinations****									
Endocrine, targeted, and chemotherapy									
NL	434 (4.0%)	11.5 (7.4, 16.9)	58 (13.4%)	313 (72.2%)	63 (14.5%)	8.7 (5.9, 13.4)	276.8	7.6 (3.9, 9.2)	3 (0.7%)
USA	535 (4.9%)	10.5 (6.2, 15.1)	64 (12.0%)	395 (73.8%)	76 (14.2%)	9.5 (6.6, 16)	305.0	6.8 (3.2, 8.2)	18 (3.4%)
Endocrine and chemotherapy									
NL	3874 (35.8%)	5.0 (3.6, 8.6)	269 (6.9%)	3035 (78.3%)	570 (14.7%)	19.9 (11.5, 27.7)	1249.2	3.9 (1.9, 4.6)	453 (11.7%)
USA	6055 (56.0%)	3.6 (2.1, 6.3)	313 (5.2%)	493 (81.3%)	819 (13.5%)	27.6 (15.8, 47.4)	1436.4	2.8 (1.1, 3.3)	2434 (40.2%)
Targeted and chemotherapy									
NL	156 (1.4%)	15.1 (13.1, 18.4)	26 (16.5%)	91 (58.4%)	39 (25.0%)	6.6 (5.4, 7.6)	179.0	13.6 (10.3, 15.2)	0 (0.0%)
USA	161 (1.5%)	15.5 (13.2, 18.5)	27 (16.6%)	93 (57.9%)	41 (25.6%)	6.4 (5.4, 7.6)	185.5	13.6 (10.4, 15.2)	0 (0.0%)

Data are given as median (IQI), unless specified otherwise. *Endocrine, targeted, and/or chemotherapy. **Total BCSS-gain from all AST in the combination therapy (monotherapy is not included). ***Contribution of AST subtype to the combination therapy. ****No patients are recommended monotargeted therapy or the combination of endocrine and targeted therapy according to the guidelines, therefore, these rows are omitted

*AST* adjuvant systemic therapy, *NL* Netherlands, *USA* United States of America, *BCSS* breast cancer-specific survival, *NNT* number needed to treat, *RMST* restricted mean survival time

## Discussion

In this study, we estimated the amount and distribution of expected overtreatment of administered and recommended AST in unilateral early breast cancer patients with real-world data from two national cancer registries. Actual treatment with any AST in the Netherlands is expected to save 6.4% of patients within 10 years (or an NNT of 21.4), whereas the remaining 93.6% of patients is expected to be overtreated. The largest amount of expected overtreatment was in the subgroup of patients who were treated with monoendocrine therapy: 96.7%. Overtreatment based on Dutch and USA guideline recommendations was also highest in the subgroup of monoendocrine therapy, respectively: 95.9% and 96.6%. A large proportion of patients treated with monoendocrine therapy in the Netherlands were actually also recommended chemotherapy. This may have led to an overestimation in overtreatment of the monoendocrine subgroup, and an underestimation in expected overtreatment of the endocrine and chemotherapy subgroup.

Our population-based AST survival gain estimates from AST differ from previously reported survival gain estimates based on randomized trial results, for example: the Early Breast Cancer Trialists’ Collaboration Group (EBCTCG) reported that 7.9% in patients aged < 50 years (or an NNT of 12.7) benefit from chemotherapy within 10 years, and 2.9% in patients aged 50–69 years (or an NNT of 34.5) [27], while our population-based estimates show that 7.3% of patients (or an NNT of 14.3) treated with monochemotherapy were expected to benefit from AST treatment, and 4.6% of patients (or an NNT of 27.3) who were treated with a combination of AST including chemotherapy were expected to benefit. Similarly, the EBCTCG report a 7.9% BCSS-gain after 5 years of tamoxifen (NNT is 12.7) [27], while our population-based estimates show that 3.3% (NNT is 38.5) were expected to benefit from monoendocrine therapy, and 4.9% (NNT is 23.3) were expected to benefit from an AST regimen including endocrine therapy. Although our estimates of overtreatment appear to be high, they largely agree with what can be expected from the randomized clinical trial results.

The issue of overtreatment has become increasingly recognized and efforts have been made to identify patients for whom AST can safely be omitted. Genomic assays, such as the 21-gene recurrence score [28] and the 70-gene signature [29, 30], have become a popular method to identify patients where chemotherapy can safely be omitted, particularly in ER+/HER2− breast cancer [28, 31–33]. However, de-escalation tools for endocrine therapy are less available [33, 34], even though approximately half of all newly diagnosed early breast cancer patients receive endocrine therapy. One reason why a higher overtreatment may be accepted in this subset of patients might be due to the fact that the adverse effects of endocrine therapy are generally regarded as less severe compared to targeted and chemotherapy [11]. However, patients are administered endocrine therapy for a long period of 5 to 10 years with side effects such as sexual dysfunction, cognitive and musculoskeletal problems that have a negative impact on the quality of life [35–37]. Therefore, also advancements in the personalization of endocrine therapy are valuable.

This study has several limitations. First, we did not obtain information regarding the use of genomic assays for both the Dutch and USA cohort, and was assumed to be unknown. This will have affected the analyses where treatment recommendations were based on guidelines, particularly for the USA guidelines (Supplemental Materials 1), and will have led to an overestimation of the amount of expected overtreatment from chemotherapy in ER+/HER2− breast cancer patients. However, even if available, we could not incorporate genomic risk in our estimation of expected BCSS-gain, as genomic risk is not included in the PREDICT model (e.g., PREDICT will overestimate BCSS in patients with high clinical but low genomic risk). Second, we applied the strictest interpretation of the guidelines which will have resulted in an underestimation of the overall amount of overtreatment, because these lenient recommendations generally apply to patients with favorable prognosis in whom BCSS-gain from AST is low. Third, our estimations are based on patient data and national guidelines from 2015; however, in 2020 both the Dutch and the NCCN guidelines have updated their AST recommendations. The Dutch guidelines in particular have de-escalated chemotherapy recommendations in ER+/HER2− breast cancer compared to the 2015 guidelines (based on 2020 guidelines; Supplemental Materials 4 shows the analyses using the new Dutch 2020 guidelines). No major updates were introduced for endocrine or targeted therapy. Registry data from 2015 were used as complete data from 2020, including administered treatment, was not available at time of the data request and no significant differences were expected in the distribution of clinicopathological variables between 2015 and 2020. Fourth, the estimations of survival and AST-specific 10-year BCSS-gain were calculated with the PREDICT algorithm. The use of expected survival benefit is necessary, as survival benefit from specific AST subtypes cannot directly be observed on a patient level from real-world clinical observational data. Therefore, the validity of our estimates depends on the validity of the PREDICT algorithm. PREDICT is validated in several independent cohorts [6, 15, 17], including a Dutch cohort [18], where it performed well, although PREDICT slightly underestimated survival in ER− and high-risk patients (T3, and grade 3), and overestimated survival in old patients (≥ 75 years) [18]. Additionally, it should be used with caution in patients aged < 40 years [38]. Still, PREDICT is endorsed by the Dutch guidelines and AJCC [7] to support clinical decision-making, and small under- and overestimations of survival are accepted. In that sense, the information we present in this study is also the information available to clinicians to support their clinical decision-making. Although, in 2015 the online prognostication most used was Adjuvant! Online (which has since been offline), which may have led to small differences in prognosis prediction compared to PREDICT [38]. Although genomic assays and prognostic tools have improved personal risk stratification, it remains difficult to predict recurrence in individual patients. Additionally, the PREDICT algorithm was developed and primarily validated in Western populations [4–6, 15–18, 38, 39], and there might be variation in competing risk among women from the age (for instance due to differences in region), which might further affect personal risk stratification. However, a validation study performed in Malaysia showed that PREDICT performed relatively well [40]. Fifth, we have estimated overtreatment distributions based on the survival over a 10-year horizon with BCSS- and RMST-gain. AST is expected to increase survival beyond this 10-year horizon, and patient-level measures such as risk of side effects, therapy adherence and effect on quality of life, but also societal-level measures such as cost–benefit analyses of the treatment should, ideally, also be taken into account [41]. Additionally, prevention of non-life threatening recurrences due to AST that could also affect health care costs and quality of life are also not taken into account. The results should be interpreted with caution, and taken as estimates. Our findings do not recommend a change in treatment guidelines, but highlight the need for tools to allow for further treatment selection in certain subgroups of breast cancer patients.

To conclude, the percentage of expected overtreatment in patients treated with combination AST and monochemotherapy was relatively high but in the range that can be expected from randomized clinical trial results. However, expected overtreatment in patients treated with monoendocrine therapy was high. Comparable results were observed when estimating survival benefit based on Dutch and USA guideline recommendations, however, as the USA guidelines recommended AST to a larger number of patients (with more prognostically favorable profiles), overtreatment was higher. De-escalation tools to curb overtreatment of endocrine therapy are especially relevant, as this subgroup represents the largest portion of breast cancer patients treated with AST.

## Supplementary Information

Below is the link to the electronic supplementary material.

Supplementary file1 (DOCX 224 kb)

## Data Availability

The data of patients in this article were provided by the Netherlands Cancer Registry and the Surveillance, Epidemiology, and End Results program by permission. Data will be shared on request to the corresponding author with permission of the Netherlands Cancer Registry and/or Surveillance, Epidemiology, and End Results program.
