# Supplementary material for: Population-based estimates of overtreatment with adjuvant systemic therapy in early breast cancer patients with data from the Netherlands and the USA
Source: Breast Cancer Res Treat. 2022 Mar 3;193(1):161–73. doi: 10.1007/s10549-022-06550-2 (PMC8993748; doi:10.1007/s10549-022-06550-2)
Supplement: Supplementary file 1 — Supplementary file1 (DOCX 224 kb) [file 10549_2022_6550_MOESM1_ESM.docx]

| 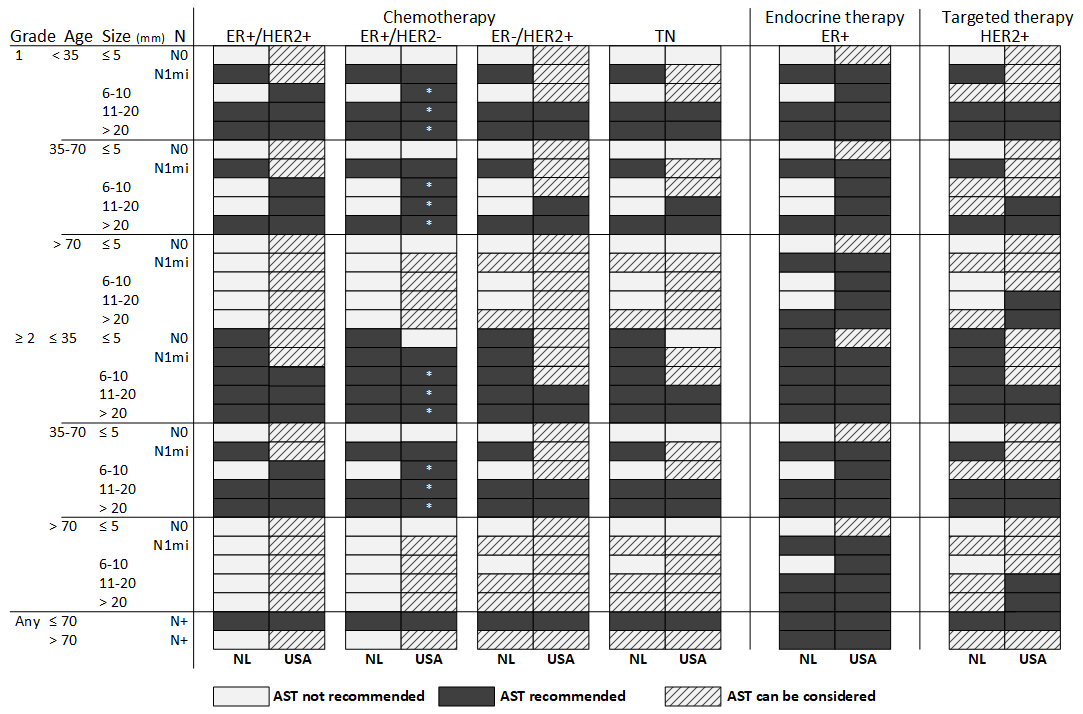 |
| --- |
| **Supplemental materials 1.** Differences in AST recommendations between the Netherlands (based on the 2015 Dutch guidelines) and the USA (based on the 2015 NCCN guidelines). *The NCCN recommends the use of the 21-gene recurrence score to support the adjuvant chemotherapy decision in early ER+/HER2- breast cancer. ER = estrogen receptor, HER2 = human epidermal growth factor-2, TN = triple negative, AST = adjuvant systemic therapy, NL = Netherlands, USA = United States of America, NCCN = National Comprehensive Cancer Network. |

| **Supplemental Materials 2.** Expected survival benefit of recommended AST regimens based on Dutch and USA guidelines in female patients surgically treated for unilateral non-metastatic breast cancer without neoadjuvant therapy in 2015 in the USA (n=45753). | | | | | | | | | |
| --- | --- | --- | --- | --- | --- | --- | --- | --- | --- |
| **AST regimen** | **Number of patients treated (% of total)** | **Median Expected 10-year BCSS-gain**  **(IQI)** | **Expected number of patient outcomes within 10 years** | | | **Expected NNT to prevent one breast cancer death in 10 years**  **(IQI)** | **Total Increased RMST in years**  **from AST within 10 years** | **Median increased RMST in months from AST within 10 years per patient (IQI)** | **Number of treated patients with <3% 10-year BCSS-gain (% of treated)** |
|  |  |  | **Benefit from AST** | **No benefit from AST, i.e., overtreatment** | |  |  |  |  |
|  |  |  | **Survived due to AST (%)** | **Survived not due to AST (%)** | **Died despite AST (%)** |  |  |  |  |
| **Any*** |  |  |  |  |  |  |  |  |  |
| NL | 27683 (60.5%) | 6.1 (3.8, 10.4) | 2280 (8.2%) | 18128 (65.5%) | 7275 (26.3%) | 16.3 (9.6, 26.2) | 11984.2 | 5.1 (2.0, 6.3) | 3993 (14.4%) |
| USA | 39169 (85.6%) | 4.4 (2.3, 8.2) | 2547 (6.5%) | 27101 (69.2%) | 9521 (24.3%) | 22.8 (12.2, 42.7) | 13070.4 | 4.0 (1.2, 5.0) | 13794 (35.2%) |
| **Chemotherapy** |  |  |  |  |  |  |  |  |  |
| Monotherapy |  |  |  |  |  |  |  |  |  |
| NL | 2256 (4.9%) | 7.6 (6.7, 9.7) | 193 (8.5%) | 1444 (64.0%) | 619 (27.5%) | 13.1 (10.3, 15) | 1324.7 | 7.0 (5.2, 7.8) | 11 (0.5%) |
| USA | 2293 (5.0%) | 7.6 (6.7, 9.7) | 195 (8.5%) | 1467 (64.0%) | 631 (27.5%) | 13.1 (10.3, 15) | 1338.1 | 6.9 (5.2, 7.8) | 26 (1.1%) |
| In combination** |  |  |  |  |  |  |  |  |  |
| NL | 18287 (40.0%) | 7.2 (4.4, 13.1) | 1795 (9.8%) | 13360 (73.1%) | 3132 (17.1%) | 13.8 (7.6, 22.6) | 9076.0 | 5.9 (2.3, 7.3) | 1049 (5.7%) |
| USA | 26725 (58.4%) | 4.9 (2.7, 10.1) | 2018 (7.6%) | 20606 (77.1%) | 4101 (15.3%) | 20.3 (9.9, 36.9) | 10012.8 | 4.5 (1.4, 5.4) | 7763 (29.0%) |
| Contribution to combination*** |  |  |  |  |  |  |  |  |  |
| NL | 18287 (40.0%) | 3.2 (1.9, 5.8) | 819 (4.5%) | 13360 (73.1%) | 4108 (22.5%) | 31.5 (17.2, 51.3) | 4119.2 | 2.7 (1.0, 3.1) | 8721 (47.7%) |
| USA | 26725 (58.4%) | 2.1 (1.2, 4.4) | 915 (3.4%) | 20606 (77.1%) | 5204 (19.5%) | 46.7 (22.7, 84.4) | 4517.3 | 2.0 (0.6, 2.3) | 16926 (63.3%) |
| **Endocrine therapy** |  |  |  |  |  |  |  |  |  |
| Monotherapy |  |  |  |  |  |  |  |  |  |
| NL | 7129 (15.6%) | 3.5 (2.5, 5.3) | 295 (4.1%) | 3334 (46.8%) | 3500 (49.1%) | 28.8 (18.7, 39.9) | 1583.5 | 2.6 (1.3, 3.2) | 2916 (40.9%) |
| USA | 10136 (22.2%) | 2.6 (1.6, 4.4) | 333 (3.3%) | 5019 (49.5%) | 4784 (47.2%) | 38.2 (22.7, 61.9) | 1719.6 | 2.2 (0.9, 2.8) | 5631 (55.6%) |
| In combination** |  |  |  |  |  |  |  |  |  |
| NL | 17527 (38.3%) | 6.9 (4.3, 12.3) | 1666 (9.5%) | 12945 (73.9%) | 2916 (16.6%) | 14.4 (8.1, 22.9) | 8167.4 | 5.5 (2.2, 6.7) | 1036 (5.9%) |
| USA | 25963 (56.7%) | 4.7 (2.6, 9.4) | 1887 (7.3%) | 20197 (77.8%) | 3879 (14.9%) | 21.0 (10.6, 37.9) | 9090.3 | 4.3 (1.3, 5.1) | 7687 (29.6%) |
| Contribution to combination*** |  |  |  |  |  |  |  |  |  |
| NL | 17527 (38.3%) | 3.8 (2.4, 6.4) | 845 (4.8%) | 12946 (73.9%) | 3736 (21.3%) | 26.5 (15.6, 41.3) | 4220.6 | 2.9 (1.3, 3.5) | 6750 (38.5%) |
| USA | 25963 (56.7%) | 2.6 (1.5, 5) | 965 (3.7%) | 20196 (77.8%) | 4802 (18.5%) | 38.3 (20, 67.9) | 4720.4 | 2.2 (0.8, 2.7) | 14524 (55.9%) |
| **Targeted therapy****** |  |  |  |  |  |  |  |  |  |
| In combination** |  |  |  |  |  |  |  |  |  |
| NL | 2835 (6.2%) | 14.9 (11, 21.5) | 476 (16.8%) | 1764 (62.2%) | 595 (21.0%) | 6.7 (4.6, 9) | 2682.3 | 11.2 (5.9, 14.3) | 23 (0.8%) |
| USA | 3400 (7.4%) | 13.4 (7, 19.5) | 514 (15.1%) | 2216 (65.2%) | 670 (19.7%) | 5.9 (3.5, 8.3) | 2861.3 | 10.0 (4.4, 13.0) | 98 (2.9%) |
| Contribution to combination*** |  |  |  |  |  |  |  |  |  |
| NL | 2835 (6.2%) | 3.9 (2.3, 6.1) | 130 (4.6%) | 1764 (62.2%) | 941 (33.2%) | 25.3 (16.3, 44) | 736.1 | 3.1 (1.2, 4.3) | 1087 (38.3%) |
| USA | 3400 (7.4%) | 3.2 (1.7, 5.6) | 138 (4.1%) | 2216 (65.2%) | 1046 (30.8%) | 31.1 (17.7, 59.2) | 775.0 | 2.7 (0.9, 3.9) | 1625 (47.8%) |
| **Specific combinations****** |  |  |  |  |  |  |  |  |  |
| Endocrine, targeted, and chemotherapy |  |  |  |  |  |  |  |  |  |
| NL | 2078 (4.5%) | 14.4 (9.4, 22) | 347 (16.7%) | 1352 (65.1%) | 379 (18.2%) | 6.9 (4.5, 10.6) | 1773.8 | 10.2 (4.9, 12.7) | 22 (1.1%) |
| USA | 2636 (5.8%) | 12.1 (7.1, 19.3) | 383 (14.5%) | 1804 (68.4%) | 449 (17.0%) | 8.2 (5.1, 14) | 1938.8 | 8.9 (3.7, 11.0) | 100 (3.8%) |
| Endocrine and chemotherapy |  |  |  |  |  |  |  |  |  |
| NL | 15442 (33.8%) | 6.3 (4.2, 10.9) | 1317 (8.5%) | 11589 (75.0%) | 2536 (16.4%) | 15.8 (9.2, 23.9) | 6393.7 | 4.9 (2.2, 5.8) | 1013 (6.6%) |
| USA | 23311 (50.9%) | 4.4 (2.5, 8.2) | 1502 (6.4%) | 18381 (78.9%) | 3428 (14.7%) | 22.9 (12.2, 40.5) | 7151.4 | 3.7 (1.3, 4.4) | 7584 (32.5%) |
| Targeted and chemotherapy |  |  |  |  |  |  |  |  |  |
| NL | 749 (1.6%) | 15.6 (13.4, 20.3) | 127 (17.0%) | 408 (54.5%) | 214 (28.5%) | 6.4 (4.9, 7.5) | 908.5 | 14.3 (10.6, 17.3) | 0 (0.0%) |
| USA | 756 (1.7%) | 15.7 (13.5, 20.5) | 130 (17.1%) | 406 (53.7%) | 220 (29.1%) | 6.4 (4.9, 7.4) | 922.5 | 14.4 (10.6, 17.4) | 0 (0.0%) |
| Data are given as median (IQI), unless specified otherwise. *Endocrine, targeted, and/or chemotherapy. **Total BCSS-gain from all AST in the combination therapy (monotherapy is not included). ***Contribution of AST-subtype to the combination therapy. ****No patients are recommended monotargeted therapy or the combination of endocrine and targeted therapy according to the guidelines, therefore, these rows are omitted. AST = adjuvant systemic therapy, NL = Netherlands, USA = United States of America, BCSS = breast-cancer specific survival, NNT = number needed to treat, RMST = restricted mean survival time. | | | | | | | | | |

| 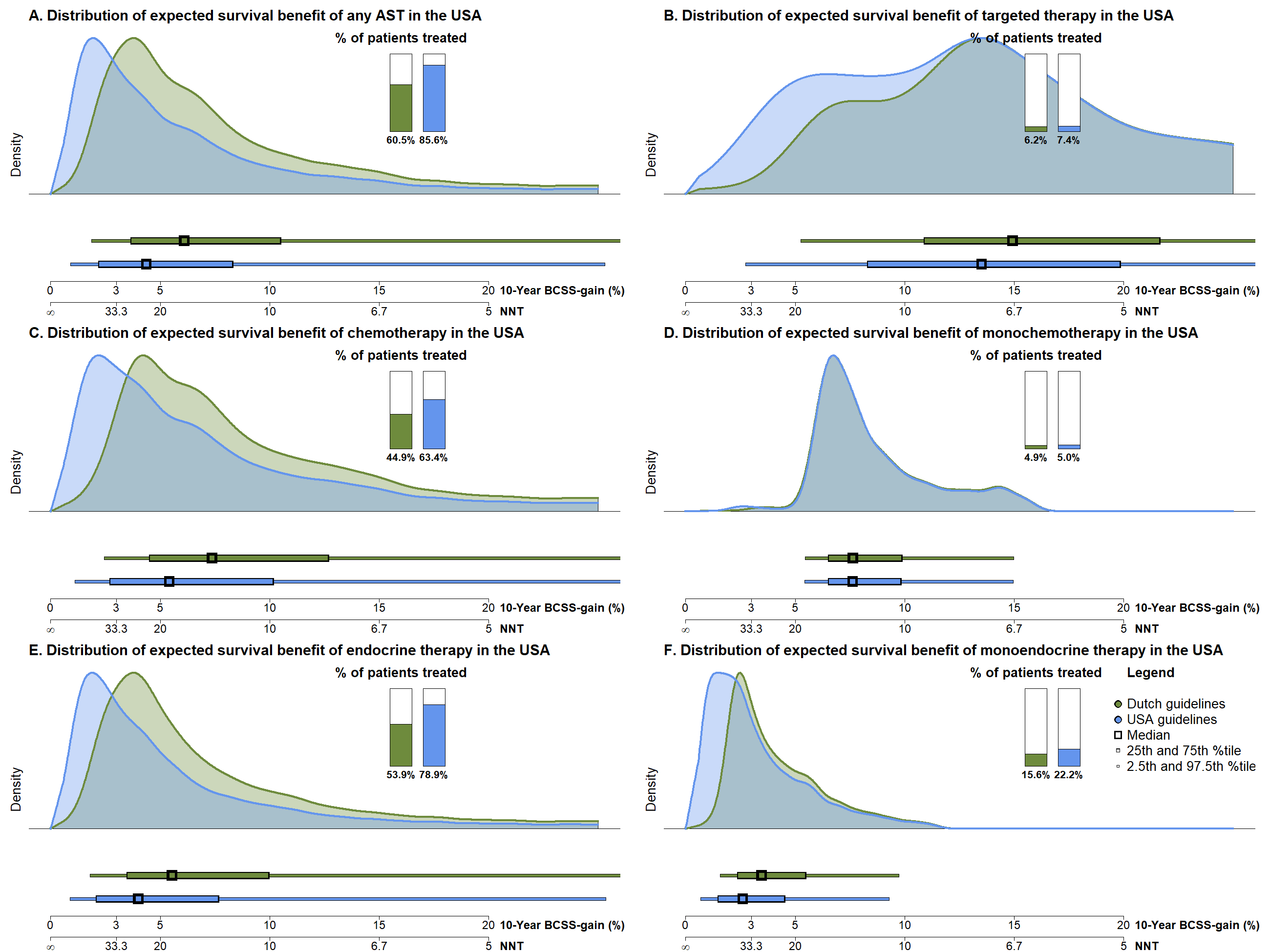 |
| --- |
| **Supplemental Materials 3.** The distribution of expected 10-year BCSS-gain and NNT for all patients in the USA who were recommended AST based on the Dutch and USA guidelines for any AST (A), targeted therapy (B), chemotherapy (C), monochemotherapy (D), endocrine (E) and monoendocrine therapy (F). For the overall AST subgroups (i.e. A, B, C, and E) the total BCSS-gain is aggregated from each received or recommended AST, e.g. chemotherapy (C) shows the distribution of total BCSS-gain of the entire registered or recommended AST regimen (including targeted and/or endocrine therapy) of patients that received chemotherapy (including monochemotherapy). Note that guidelines do not recommend treatment with monotargeted therapy, therefore, the distribution is unavailable. AST = adjuvant systemic treatment, BCSS = breast cancer-specific survival, NNT = number needed to treat, USA = United States of America. |

| **Supplemental Materials 4.** Expected survival benefit of recommended AST regimens based on 2015 and 2020 Dutch in female patients surgically treated for unilateral non-metastatic breast cancer without neoadjuvant therapy in 2015 in the Netherlands (n=10810). | | | | | | | | | |
| --- | --- | --- | --- | --- | --- | --- | --- | --- | --- |
| **AST regimen** | **Number of patients treated (% of total)** | **Median Expected 10-year BCSS-gain**  **(IQI)** | **Expected number of patient outcomes over a 10-year horizon** | | | **Expected NNT to prevent one breast cancer death in 10 years**  **(IQI)** | **Total increased RMST in years from AST within 10 years (years)** | **Median increased RMST in months from AST within 10 years per patient (IQI)** | **Number of treated patients with <3% 10-year BCSS-gain (% of treated)** |
|  |  |  | **Benefit from AST** | **No benefit from AST, i.e., overtreatment** | |  |  |  |  |
|  |  |  | **Survived due to AST (%)** | **Survived not due to AST (%)** | **Died despite AST (%)** |  |  |  |  |
| **Any*** |  |  |  |  |  |  |  |  |  |
| 2015 Guidelines | 6782 (62.7%) | 5.3 (3.5, 8.5) | 470 (6.9%) | 4678 (69.0%) | 1634 (24.1%) | 19.0 (11.7, 28.9) | 2378.7 | 4.2 (1.8, 5.3) | 1155 (17.0%) |
| 2020 Guidelines | 6646 (61.5%) | 6.5 (3.6, 8.7) | 444 (6.7%) | 4569 (68.8%) | 1633 (24.6%) | 18.4 (11.4, 27.9) | 2265.9 | 4.1 (1.2, 5.4) | 2313 (34.8%) |
| **Chemotherapy** |  |  |  |  |  |  |  |  |  |
| Monotherapy |  |  |  |  |  |  |  |  |  |
| 2015 Guidelines | 554 (5.1%) | 7.3 (6.4, 8.8) | 44 (8.0%) | 370 (66.7) | 140 (25.3%) | 13.8 (11.3, 15.7) | 299.9 | 6.5 (5.0, 7.0) | 0 (0.0%) |
| 2020 Guidelines | 492 (4.6%) | 7.5  (6.4, 9) | 40 (8.2%) | 325 (66.0%) | 127 (25.9%) | 13.3  (11.1, 15.5) | 273.2 | 6.6 (5, 7.3) | 0 (0.0%) |
| In combination** |  |  |  |  |  |  |  |  |  |
| 2015 Guidelines | 4471 (41.4%) | 5.7 (3.8, 10.2) | 354 (7.9%) | 3444 (77.0%) | 673 (15.1%) | 17.5 (9.8, 26.5) | 1705.1 | 4.6 (1.9, 5.5) | 456 (10.2%) |
| 2020 Guidelines | 2856 (26.4%) | 8.7  (6.1, 13.2) | 300 (10.5%) | 2058 (72.1%) | 498 (17.4%) | 11.3 (17.4, 16.1) | 1485.7 | 6.2 (3.2, 7.6) | 84 (2.9%) |
| Contribution to combination*** |  |  |  |  |  |  |  |  |  |
| 2015 Guidelines | 4471 (41.4%) | 2.5 (1.7, 4.4) | 160 (3.6%) | 3444 (77.0%) | 867 (19.4%) | 40.2 (22.7, 60.1) | 772.9 | 2.1 (0.8, 2.3) | 2639 (59.0%) |
| 2020 Guidelines | 2856 (26.4%) | 3.8  (2.7, 6.1) | 138 (4.8%) | 2058 (72.1%) | 660 (23.1%) | 26.0  (16.4, 37.3) | 684.0 | 2.9 (1.4, 3.3) | 959 (33.6%) |
| **Endocrine therapy** |  |  |  |  |  |  |  |  |  |
| Monotherapy |  |  |  |  |  |  |  |  |  |
| 2015 Guidelines | 1751 (16.2%) | 3.5 (2.4, 5.3) | 71 (4.1%) | 861 (49.1%) | 819 (46.8%) | 28.6 (18.9, 41.2) | 373.8 | 2.6 (1.3, 3.1) | 695 (39.7%) |
| 2020 Guidelines | 3284 (30.4%) | 3.6  (2.9, 4.5) | 102 (3.1) | 2178 (66.3%) | 1004 (30.6%) | 27.5  (22.3, 34.4) | 507.0 | 1.9 (1.0, 2.0) | 2225 (67.8%) |
| In combination** |  |  |  |  |  |  |  |  |  |
| 2015 Guidelines | 4312 (39.9%) | 5.5 (3.7, 9.5) | 328 (7.6%) | 3351 (77.7%) | 633 (14.7%) | 18.1 (10.5, 26.8) | 1526.1 | 4.2 (1.9, 5.1) | 456 (10.6%) |
| 2020 Guidelines | 2567 (23.7%) | 8.6  (6.2, 12.8) | 267 (10.4%) | 1861 (72.5%) | 439 (17.1%) | 11.6  (7.8, 16.2) | 1267.9 | 5.9 (3.2, 7.0) | 18 (0.7%) |
| Contribution to combination*** |  |  |  |  |  |  |  |  |  |
| 2015 Guidelines | 4312 (39.9%) | 3.0 (2.1, 5) | 172 (4.0%) | 3351 (77.7%) | 790 (18.3%) | 33.5 (19.8, 48.1) | 807.4 | 2.2 (1.1, 2.7) | 2167 (50.3%) |
| 2020 Guidelines | 2567 (23.7%) | 4.6  (3.4, 6.5) | 137 (5.3%) | 1861 (72.5%) | 569 (22.2%) | 21.6 (15.2, 29.7) | 661.4 | 3.1 (1.8, 3.7) | 435 (16.9%) |
| **Targeted therapy****** |  |  |  |  |  |  |  |  |  |
| In combination** |  |  |  |  |  |  |  |  |  |
| 2015 Guidelines | 592 (5.5%) | 13.0 (9.1, 17.4) | 84 (14.2%) | 406 (68.5%) | 102 (17.3%) | 7.7 (5.7, 10.9) | 455.9 | 9.2 (4.9, 11.7) | 3 (0.5%) |
| 2020 Guidelines | 725 (6.7%) | 11.7  (5.9, 16.3) | 92 (12.7%) | 508 (70.1%) | 125 (17.2%) | 7.0 (4.1, 9.8) | 501.4 | 8.3 (3.5, 10.9) | 66 (9.1%) |
| Contribution to combination*** |  |  |  |  |  |  |  |  |  |
| 2015 Guidelines | 592 (5.5%) | 3.0 (1.8, 5.1) | 22 (3.8%) | 406 (68.5%) | 164 (27.7%) | 33.6 (19.7, 54.4) | 124.8 | 2.5 (0.9, 3.7) | 297 (50.2%) |
| 2020 Guidelines | 725 (6.7%) | 2.7  (1.5, 4.8) | 25 (3.5%) | 508 (70.1%) | 192 (26.4%) | 37.3 (20.8, 68.1) | 140.3 | 2.3 (0.8, 3.5) | 395 (54.5%) |
| **Specific combinations****** |  |  |  |  |  |  |  |  |  |
| Endocrine, targeted, and chemotherapy |  |  |  |  |  |  |  |  |  |
| 2015 Guidelines | 434 (4.0%) | 11.5 (7.4, 16.9) | 58 (13.4%) | 313 (72.2%) | 63 (14.5%) | 8.7 (5.9, 13.4) | 276.8 | 7.6 (3.9, 9.2) | 3 (0.7%) |
| 2020 Guidelines | 437 (4.0%) | 11.6 (7.5, 17) | 59 (13.6%) | 313 (71.5%) | 65 (15.0%) | 8.6 (5.9, 13.2) | 283.5 | 7.7 (3.9, 9.3) | 1 (0.2%) |
| Endocrine and chemotherapy |  |  |  |  |  |  |  |  |  |
| 2015 Guidelines | 3874 (35.8%) | 5.0  (3.6, 8.6) | 269 (6.9%) | 3035 (78.3%) | 570  (14.7%) | 19.9 (11.5, 27.7) | 1249.2 | 3.9 (1.9, 4.6) | 453 (11.7%) |
| 2020 Guidelines | 2126 (19.7%) | 8.2 (6, 11.6) | 207 (9.8%) | 1546 (72.7%) | 373 (17.5%) | 12.2 (8.6, 16.5) | 984.4 | 5.5 (3.2, 6.4) | 17 (0.8%) |
| Targeted and chemotherapy |  |  |  |  |  |  |  |  |  |
| 2015 Guidelines | 156 (1.4%) | 15.1  (13.1, 18.4) | 26 (16.5%) | 91 (58.4%) | 39  (25.0%) | 6.6 (5.4, 7.6) | 179.0 | 13.6 (10.3, 15.2) | 0 (0.0%) |
| 2020 Guidelines | 285 (2.6%) | 12.5  (3.9, 16.1) | 32 (11.4%) | 194 (68.0%) | 59 (20.6%) | 7.4 (5.4, 13.9) | 217.9 | 9.0 (2.1, 12.8) | 64 (22.5%) |
| Data are given as median (IQI), unless specified otherwise. *Endocrine, targeted, and/or chemotherapy. **Total BCSS-gain from all AST in the combination therapy. ***Contribution of AST-subtype to the combination therapy. ****No patients are recommended monotargeted therapy or the combination of endocrine and targeted therapy according to the guidelines, therefore, these rows are omitted. AST = adjuvant systemic therapy, BCSS = breast-cancer specific survival, NNT = number needed to treat, RMST = restricted mean survival time. | | | | | | | | | |
